# The molecular mechanism of thrombospondin family members in cardiovascular diseases

**DOI:** 10.3389/fcvm.2024.1337586

**Published:** 2024-03-07

**Authors:** Heng Pan, Xiyi Lu, Di Ye, Yongqi Feng, Jun Wan, Jing Ye

**Affiliations:** ^1^ Department of Cardiology, Renmin Hospital of Wuhan University, Wuhan, China; ^2^ Cardiovascular Research Institute, Wuhan University, Wuhan, China; ^3^ Hubei Key Laboratory of Cardiology, Wuhan, China

**Keywords:** cardiovascular disease, thrombospondin, cartilage oligomeric matrix protein, myocardial remodeling, vascular remodeling

## Abstract

Cardiovascular diseases have been identified as vital factors in global morbidity and mortality in recent years. The available evidence suggests that various cytokines and pathological proteins participate in these complicated and changeable diseases. The thrombospondin (TSP) family is a series of conserved, multidomain calcium-binding glycoproteins that cause cell-matrix and cell-cell effects via interactions with other extracellular matrix components and cell surface receptors. The TSP family has five members that can be divided into two groups (Group A and Group B) based on their different structures. TSP-1, TSP-2, and TSP-4 are the most studied proteins. Among recent studies and findings, we investigated the functions of several family members, especially TSP-5. We review the basic concepts of TSPs and summarize the relevant molecular mechanisms and cell interactions in the cardiovascular system. Targeting TSPs in CVD and other diseases has a remarkable therapeutic benefit.

## 1 Introduction

Currently, cardiovascular disease (CVD) is a vital cause of human disability and death in both underdeveloped and developed regions (1). In addition to the large cost of treatment during disease onset, the expense of complications and recovery poses a severe burden to families and communities. Although many effective drugs and advanced technologies have been used in clinical practice in recent years, CVD incidence still correlates with poor overall survival and prognosis.

Thrombospondin (TSP) is a matricellular protein that can be secreted by many cell types and is widely distributed in various organs and tissues (2). The TSP family includes five extracellular, conserved matricellular proteins in mammals. The TSP family is divided into two subgroups (Group A and Group B) based on their structure. Group A includes TSP-1 and TSP-2, which are trimers. Group B is composed of TSP-3, TSP-4, and TSP-5, which are pentameric proteins (3). The expression of TSP is low under normal physiological conditions but increases in response to damage, and TSP is subsequently involved in tissue repair or deterioration (4). TSP has a multimeric structure that allows it to bind calcium, cell-surface proteins, bioactive effectors, and other extracellular matrix (ECM) proteins. TSP has many complex and variable functions, such as regulating wound healing, angiogenesis, and tissue remodeling. In the cardiovascular system, TSP participates in regulating vasomotor function, adjusting cell apoptosis and growth, reacting to cardiovascular injuries, and affecting the structural integrity of the heart and blood vessels (5). Many studies have revealed a close link between TSP and CVD. Group A TSPs and TSP-4 from group B are the most thoroughly studied TSPs. We subsequently summarize the functions of TSPs, especially TSP-5, in cardiovascular pathological processes and update the role of group B TSPs in CVD treatment.

CVD and cancer are considered two mutually independent diseases. Along with the increase in cancer survivors and the application of new therapeutic strategies for distinct cancers, patients who suffer from cancer often have a greater risk of cardiovascular complications than individuals in general. Thus, a new discipline called cardio-oncology (6) has gained attention from clinicians and cardiologists. However, the latent links between CVD occurrence and consequential carcinoma have been less investigated. Studies have shown that people with cardiovascular disorders have a greater risk of cancer than does the general public, which is called “reverse cardio-oncology (7, 8)”. This argument is based on the shared risk factors and pathogenic mechanisms involved in these two diseases (9). For example, alcoholism, obesity, and diabetes mellitus are the same causes of CVD and cancer (7, 10). The TSP family also plays a role in shared pathogenic mechanisms and pathways. TSP-4 is involved in CVD and regulates several different cancers, such as colon cancer and prostate cancer, and the effects of TSP-4 on cancer cells are complex and opposite. Overexpression of TSP-4 inhibited the tumor growth of colorectal cancers, but a lack of TSP-4 in prostate cancer cells reduced their invasion and migration (11, 12). In addition, the clinical application of TSPs is closely related to cancer treatment. In this review, we focused on the role of TSPs in the cardiovascular system in two subgroups based on the pathological mechanism, as well as other closely linked diseases and clinical treatment strategies.

## 2 The structure of the TSP family

**Figure 1:**
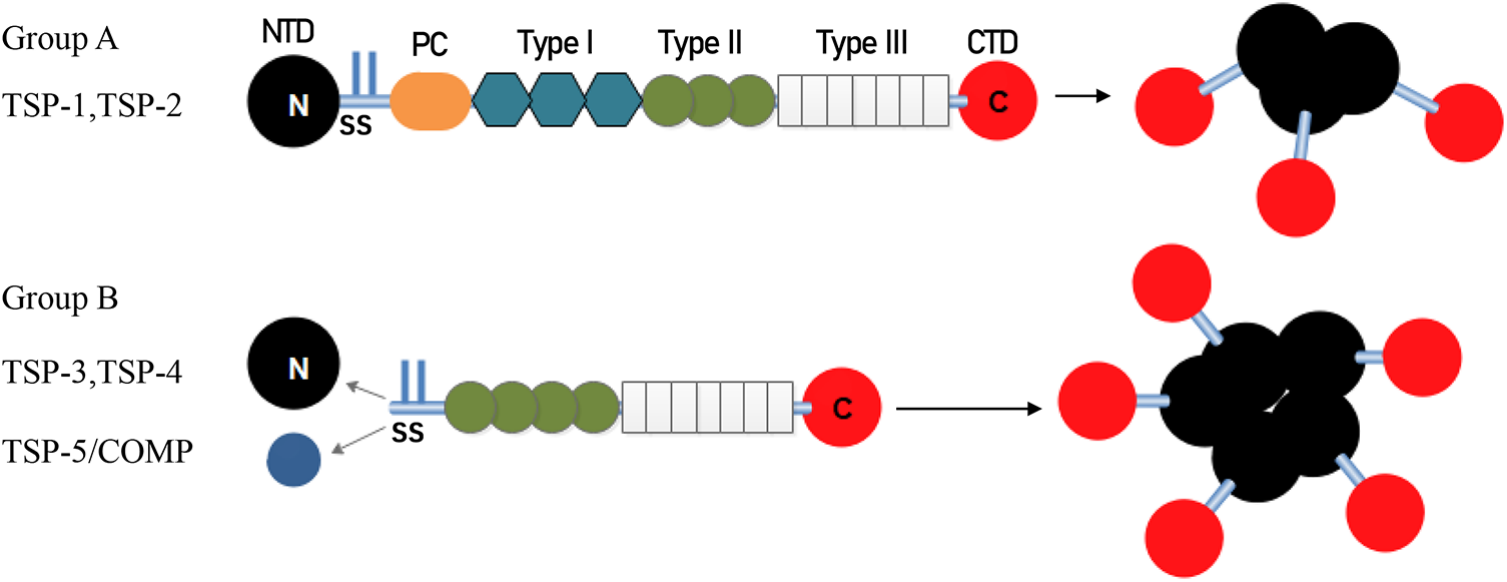
Architecture and oligomerization status of group A and group B TSP. TSP-1 and TSP-2 (Group A) assemble into trimeric structure. TSP-3, 4 and 5 (Group B) assemble into pentamers. TSP, thrombospondin; NTD, N-terminal domain; SS, disulfide bonds; PC, homologous procollagen region, Type I, thrombospondin type 1 domains; Type II, epidermal growth factor-like domains; Type III, thrombospondin type 3 repeats; CTD, C-terminal domain.

The structure of representative TSP polypeptides is shown in Figure 1. TSP subunits have a highly conserved carboxy terminus that binds to many epidermal growth factor (EGF)-like repeats, which are called TSP type 2 and are linked to seven TSP type 3 repeats and a globular C-terminal domain (CTD). The CTD shares common extracellular, calcium-binding, and cell membrane-binding properties (13). In addition to an N-terminal domain (NTD), a procollagen homology domain (PC), and an oligomerization sequence, group A TSPs also have type 1 domains (TSRs), which are composed of three properdin-like repeats called antiangiogenic regions that are involved in modulating antiangiogenic functions and accelerating cell attachment (3). In contrast, group B has no TSRs, and the procollagen homology domain contains four (group A is three) type 2 repeats instead. Inter-subunit disulfide bonds are formed between cysteine residues adjacent to the carboxyl end of the heptad fold repeat sequence in trimer TSP or between the carboxyl end of the pentamer TSP to stabilize the low polymerization of TSP. Because of the variable region, group B has different effects on different tissues (14).

Due to the availability of each domain, TSPs can interact with diverse surface receptors and proteins. For example, the C-terminal domain contains a CD47-binding site, EGF-like domains can bind to integrins and Ca^2+^, TSRs are necessary for binding transforming growth factor (TGF)-β and CD36, and heparin and other integrins bind to the N-terminal domain (15). We analyzed the role of TSP in CVDs according to different receptors, signaling pathways, and immune cells involved.

## 3 Group A TSPs

### 3.1 Interacting receptors

As stated above, different domains of the TSP can interact with corresponding receptors, including integrins, CD36 and CD47. Integrins are a class of glycosylated, heterodimeric transmembrane receptors that consist of α and β subunits. The interaction of cardiac ECM with specific cell surface integrins is the foundation of cardiomyocyte maturation and remodeling (16).

Moreover, the interaction between TSPs and integrins participates in vascular remodeling and pathology. TSP-1 and TSP-2 are regarded as potent endogenous antiangiogenic proteins that directly affect CD36, CD47, and integrins. Vascular cells express various integrins that bind to TSP-1, including *α*_Iib_*β*_3_, *α*_v_*β*_3_, and *α*_5_*β*_1_ (17). The N-termini of TSP-1 and TSP-2 can be recognized by *α*_3_*β*_1_, *α*_4_*β*_1_, and *α*_6_*β*_1_ to mediate the adhesion of vascular ECs (18). The binding of integrin *α*_3_*β*_1_ to TSP-1 promotes EC migration, cell motility, and antiangiogenic effects (19). In another study, TSP-1 was shown to bind to *α*v*β*1 and regulate the nuclear shuttling of Yes-associated protein in response to mechanical stress-induced vascular injury, leading to dynamic remodeling of the aorta in mice (20).

### 3.2 Transforming growth factor-β (TGF-β)

The TGF-β superfamily has a significant role in inducing tissue fibrosis and inflammation. Type I repeats of TSP can interact with latent TGF-β. For instance, TGF-β signaling enables the ERK1/2 pathway to activate fibroblasts and induce interstitial fibrosis in the aging course (21). The TGF-β family is mainly classified into the TGF-β1, -2, and -3 subfamilies. One of the downstream mediators of TGF-β1, connective tissue growth factor (CTGF), also plays an important role in fibrosis.

TSP-1 is a major mediator of TGF-β activation, increasing CTGF and collagen levels and the accumulation of extracellular matrix proteins (22). All of these factors stimulate and are hallmarks of tissue fibrosis. Increased TSP-1 in intermittent hypoxia (IH) patients and mice, which activates the TGF-β pathway via JAK2/STAT3/TSP-1 signaling, has a significant effect on IH-induced fibroblast activation and cardiac fibrosis (23). A TSP-1 antagonist that blocks TGF-β activation can reverse myocardial fibrosis and ensure left ventricular function in hypertensive diabetic rats (24). TSP-1 acted as a protective factor by maintaining fibroblast function and matrix metabolism in pressure-overloaded myocardium. TSP-1 is also downstream of the TGF-β signaling pathway and has been identified as a regulator of microtube formation in glioblastoma (25). In addition to the heart, as a result of TSP-1/TGF-β pathway upregulation, collagen deposition and extensive fibrosis in the arterial wall led to arteriosclerosis, while in TSP-1 knockout (KO) mice or mice treated with TSP-1 antagonist, arterial collagen, CTGF, and arterial stiffness were decreased (26). *Ying Xia* et al. reported that deletion of TSP-1 disrupted TGF-β signaling, leading to impaired myofibroblast differentiation and decreased collagen expression (27).

TSP-2 can bind to latent TGF-β but cannot activate the TGF-β signal and is regarded as a competitive binding factor to TSP-1 in CVD. However, in the cancer microenvironment, a high level of TSP-2 produced by cancer-associated fibroblasts is activated via the TGF-β1/Smad2/3 pathway, binds to integrin *α*_v_β_3_/CD36 and activates the MAPK pathway in cancer cells to promote tumor growth and adhesion (28).

Regarding injury to other organs, TSP-1 is highly expressed in patients with sepsis-induced acute kidney injury (AKI). The transcription factor USF2 activates TSP-1 to activate the TGF-β/NLRP3/Caspase-1 signaling pathway, resulting in promotion of the oxidative stress response and stimulation of pyroptosis in sepsis-induced AKI (29). TSP-1 deficiency protects mice against sepsis-induced AKI by decreasing the expression of inflammatory and apoptosis-promoting cytokines, such as the NLRP3 inflammasome, caspase-1, IL-1β, and IL-18, which increase cell viability and partially reverse cell pyroptosis. TSP-1 deletion reduces TGF-β signaling and protects against renal fibrosis in a high-fat diet mouse model (30). In another study, interstitial macrophages secreted TSP-1 after hypoxia exposure, and pathological TSP-1 promoted TGF-β activation and Rho-kinase-mediated vasoconstriction in mice, resulting in pulmonary hypertension (31).

### 3.3 Endoplasmic reticulum stress

The endoplasmic reticulum (ER) is a large protein processing and transporting region. Mistaken ER protein folding leads to the accumulation of unfolded and misfolded proteins. This disturbance of ER homeostasis initiates the protective stress response and is known as the unfolded protein response (UPR) or ER stress (32). Protein kinase R-like ER kinase (PERK), activating transcription factor 6α (ATF6α), and inositol requiring enzyme 1 alpha (IRE1α) are three primary sensors that strengthen protein folding under ER stress (33). ER stress is a double-edged sword that can restore cell homeostasis and may lead to cell defects (32). In TSP-1-overexpressing transgenic mice, TSP-1 binds to PERK and induces the downstream factor ATF4, thus activating ER stress and inducing autophagy-mediated lethal cardiac atrophy (34).

However, for the other member of group A, TSP-2, further research is needed to reveal the possible links between TSP-2 and ER stress.

### 3.4 Nitric oxide (NO) signaling

Nitric oxide (NO) is a vital physiological regulator of vasomotion and blood flow. TSP-1 affects the proliferation, migration, and angiogenesis of SMCs, ECs, and platelets (PLTs) by regulating NO signaling (22, 35). TSP-1 binds to the cell surface proteins CD47 and CD36 and subsequently participates in the activity of endothelial nitric oxide synthase (eNOS) (36, 37). Moreover, the combination of TSP-1 with CD47 or CD36 can inhibit the SMC cyclic guanosine monophosphate pathway in the presence of a low dose of NO (NO/cGMP) (38), exerting antiangiogenic effects and activating apoptosis in microvascular ECs, which leads to failed endothelial tubule formation and directly affects other antiangiogenic activities. Studies have shown that, compared with CD36, CD47 has a greater affinity for TSP-1 and inhibits NO signaling (39). Through binding to CD47, TSP-1 inhibits eNOS activation and arterial relaxation, manifesting as a blood pressure booster agent (40).

TSP-1 engages the receptor CD47 of ECs to mediate cell phenotypic transformation, and the activation of CD47 inhibits the bioavailability of VEGF (41, 42), as observed in lymphatic endothelial cells, resulting in AKT-eNOS signaling activation and NO reduction (43). Thus, silencing CD47 blocks lymphangiogenesis and atherosclerotic lesion formation (43). Deletion of TSP-1 protects against inflammatory lesion development and vascular smooth muscle cell (VSMC) phenotypic transition in leptin-induced atherogenesis (44). TSP-1 can also suppress the NO signaling pathway by interacting with integrin to regulate platelet aggregation (39).

TSP-2 seems to be an NO target, and suppressing TSP-2 relieves the eNOS-knockout phenotype without restoring NO signaling in mice with injury- and ischemia-induced angiogenesis (45).

### 3.5 Matrix metalloproteinase

Matrix metalloproteinases, such as matrix metallopeptidase (MMP)-2, MMP-3, and MMP-9, participate in tissue remodeling by binding to TSP type I repeats (27).

TSP-1 deficiency increases myocardial MMP-3 and MMP-9 activation under pressure overload, resulting in early cardiac hypertrophy and late dilation. During remodeling of the diabetic heart, TSP-1 deletion increases MMP-2 and MMP-9 activation to degrade collagen and inhibit fibroblast function (46).

TSP-2 acts as a key regulator of cardiac matrix integrity, is required for the myocardium to respond to pressure overload, and plays a role in regulating MMP activity (47). TSP-2 plays a protective role in age-related cardiomyopathy by activating the Src/Akt survival pathway, decreasing inflammation and MMP-2 activity, and maintaining collagen crosslinking (48). High levels of MMP-2 (49) and MMP-9 (50) may cause local disruption of the myocardial matrix, leading to cardiac rupture and dilatation after Ang II infusion in TSP-2-deficient mice (47). Enhanced matrix destruction is shown in the heart of doxorubicin (DOX)-induced TSP-2 KO mice and is accompanied by increased levels of MMP-2 (51). A lack of TSP-2 increases the levels of MMPs in the extracellular matrix and helps with the degradation of fibrillar collagen, thus reducing fibrosis around cardiac cell grafts (52).

MMPs also take part in vascular physiology. TSP-1 can be hydrolyzed into proteolytic fragments, and the different fragments exert pro- or antiangiogenic effects by activating the MAPK pathway to mediate the MMP/tissue inhibitor of metalloproteinase (TIMP) balance (53). TSP-2 inhibits angiogenesis by regulating EC function and modulating MMP-2 and MMP-9 (54).

### 3.6 Reactive oxygen species

Reactive oxygen species (ROS) are associated with senility and injury, regulating myocardial metabolism and contributing to vascular dystonia (55). The family of nicotinamide adenine dinucleotide phosphate (NADPH) oxidase (Nox) isozymes is the main source of ROS. Nox-4-based NADPH oxidases are the main source of ROS in the vasculature, and downregulation of Nox4 in SMCs inhibits neointimal hyperplasia by decreasing TSP-1 and suppressing SMC proliferation (56). Hypoxia-responsive TSP-1 mediates the critical event in pulmonary hypertension. The level of TSP-1 was increased in hypoxia-induced human pulmonary artery SMCs, and TSP-1-stimulated Nox4 expression was enhanced, causing SMC proliferation and high blood pressure (57). O_2_^−^ is the first product induced by Nox and rapidly changes into stable H_2_O_2_. Moreover, excess accumulation of O_2_^−^ disrupts the balance of O_2_^−^/H_2_O_2_, resulting in the impairment of coronary arteriolar vasodilation and heart ischemia (58, 59).

In diabetic conditions, TSP-1 binds to the cell-surface receptor CD47 and significantly increases Nox1-mediated ROS and O_2_^−^ production, causing endothelial senescence and vascular dysfunction (60–62), while TSP-2 plays an antiangiogenic role. Increasing NADPH oxidase activity and oxidative stress induce the production of TSP-2, which induces bone marrow-derived angiogenic cell dysfunction and vascular impairment (63). The TSP-1/CD47 axis also activates signal-regulatory protein-α, another kind of cell-surface receptor of inflammatory cells, increasing O_2_^−^ production and promoting renal ischemia‒reperfusion injury (64). H_2_O_2_ can induce vasodilation through the release of prostaglandin E2 and calcium-activated potassium channel-related SMC hyperpolarization.

### 3.7 Plasmin/plasminogen system

Activation of the plasmin/plasminogen system is necessary for regulating angiogenesis in a variety of diseases (65). A study indicated that plasminogen decreases the expression of TSP-1 and TSP-2, thereby enhancing angiogenesis in damaged brain tissue to help cells resume after ischemic stroke (66). However, no additional studies have evaluated the relationship between TSP-2 and the plasmin/plasminogen system.

The absence of plasminogen suppresses EC migration and decreases cerebrovascular density. Platelet activation releases stored TSP-1 in platelet α-granules; thus, plasma TSP-1 is significantly increased during the acute stage in ST-segment elevation myocardial infarction (STEMI) patients and decreases 1–3 days and 3 months after percutaneous coronary intervention (PCI), which might be associated with major adverse cardiac events (67).

In addition, both plasmin and TSP-1 can activate TGF-β1 signaling in the fibrotic process (68, 69). However, TGF-β1 activation is bistable: the plasmin-mediated mode is characterized by low activation, and the TSP-1-mediated mode is characterized by high activation. Interestingly, when both plasmin and TSP-1 are present, increasing plasmin can disrupt the TSP-1/TGF-β1 feedback loop and thus cause proteolytic cleavage of TSP-1 and inactivation of TGF-β1 signaling (70). This phenomenon corroborates that the activation of plasmin inhibits the expression of TSP-1.

### 3.8 Immune cells

Activation of the TSP-1/CD47 pathway plays a crucial role in the excitation and migration of T regulatory cells (Tregs) to inhibit inflammation in atherosclerosis and abdominal aortic aneurysm (AAA) (71, 72). TSP-1 and TSP-2 also interact with CD47 on the surface of T cells, which triggers T-cell apoptosis to block inflammation. In CD47-knockout mice, TSP-1- or TSP-2-knockout mice, prolonged inflammation occurs along with defects in T-cell apoptosis (73). TSP-2 is also an important regulator of T-lymphocyte migration and differentiation through its interaction with CD47 (71) in humans and mice (74). TSP-2 deficiency results in the generation of fewer Tregs and lower IL-10 and IL-10 receptor levels, thereby leading to an imbalance in the immune response and cardiac damage in Coxsackievirus group B type 3 (CVB3) virus-induced myocarditis (74, 75).

MMPs are necessary for macrophages to persist and migrate into the ECM to induce an inflammatory response in AAA (76). TSP-1 deficiency upregulates the tissue inhibitor MMP-1, which suppresses extracellular gelatinase activity and inhibits MMP activation, thus alleviating AAA development (77). TSP-1 deficiency also enhances atherosclerotic plaque maturation by accelerating inflammation, and macrophage-induced MMP-9 contributes to elastin degradation (78). TSP-1 can bind to CD47 in macrophages to enhance the inflammasome-dependent maturation of IL-1β, which promotes inflammation in response to lipopolysaccharide (LPS) (79). TSP-1 may promote proinflammatory macrophage differentiation to aggravate human aortic dissection (AD) (80). Another study showed that the overexpression of TSP-2 promotes the polarization of macrophages toward an anti-inflammatory phenotype by activating the PI3K pathway *in vitro* and attenuating LPS-induced pulmonary inflammation (70).

TSP-1 serves as a master regulator of cancer invasion (81, 82). A study showed that the expression of TSP-1 and CD47 is increased in human malignant melanoma tumor tissue, and targeting the TSP-1/CD47 pathway may preserve CD8^+^ T-cell activation, proliferation, and bioenergetics to alleviate the tumor burden (83). By the way, downregulating TSP-1 levels in skin dendritic cells effectively promoted antitumor reactions through increasing tumor-infiltrating CD4^+^ and CD8^+^ T cells. Moreover, TSP-1-knockout bone marrow-derived DCs retarded tumor growth, while targeting TSP-2 did not have the same effect (84). This finding was opposite to the antitumor angiogenesis response of TSP-1/2, indicating that systemic antitumor treatment with TSP-1 could be a double-edged sword.

## 4 Group B TSPs

### 4.1 Integrin and related signaling pathways

Group B TSPs binds to integrin to regulate physiological and pathological processes. Overexpression of TSP-3 in the mouse heart significantly damages cardiomyocyte integrity due to reduced sarcolemmal residence of integrins, such as *α*_7_*β*_1D_ and β_1_ (85). Studies have shown that the expression of TSP-5 is decreased in the hearts of DCM patients and that a lack of TSP-5 in mice spontaneously results in DCM at a young age (86). TSP-5 interacts with cardiomyocyte integrin β_1_ to maintain cardiomyocyte homeostasis, and TSP-5 or integrin β_1_ deficiency results in similar F-actin dissolution, connexin-43 defects, and spontaneous apoptosis (86).

The interaction of TSP-4 and *α*_M_β_2_ and β_3_ integrins in macrophages activates p38-MAPK signaling (87). The p38-MAPK pathway plays a pivotal role in the production of proinflammatory mediators and cytokines, as well as in endothelial-leukocyte interactions, resulting in vascular inflammation and arteriosclerosis (87–89). TSP-4 also modulates the proliferation of SMCs and ECs, thereby exerting atherogenic effects (90). Thus, TSP-4 serves as a proangiogenic factor in wound healing. TSP-4 regulates the adhesion, migration, and proliferation of EC cells by interacting with integrin *α*_2_ and gabapentin receptor *α*_2_*δ*-1 (91), resulting in a strong proangiogenic effect *in vivo* and *in vitro*.

Bone marrow-derived TSP-5 mediates atherosclerotic calcification, and a lack of TSP-5 induces the atherogenic and osteogenic phenotype in macrophages via integrin β_3_ (92). TSP-5 is involved in the chemotaxis and attachment of VSMCs and inhibits osteochondrogenic phenotypic switching in VSMCs, thereby inhibiting vascular calcification (93, 94). Increased TSP-5 levels during injury or other pathological processes exert protective effects by maintaining the contractile phenotype of VSMCs through interactions with the integrin *α*_7_β_1_ (93, 94). In addition, the C-terminus of TSP-5 binds directly to integrin *α*_5_, blocking aberrant activation of ECs in mice and hence reducing vascular inflammation and atherosclerosis (95).

### 4.2 TGF-β signal

TSP-4 and TSP-5 regulate atherosclerosis, aortic aneurysm, and other vascular tissue remodeling processes through the TGF-β signal pathway (96). Activated TGF-β1 stimulates the Smad3 pathway to upregulate endothelial TSP-4, leading to EC adhesion, migration, and proliferation (97). This strong proangiogenic function can be blocked in TSP-4 KO mice. Smad3 activation in ECs also upregulates TSP-4 expression and angiogenesis via TGF-β during tumor growth (97).

TSP-5, also known as cartilage oligomeric matrix protein (COMP), participates in the differentiation of stem cells and passaged chondrocytes and is dependent on TGF-β signaling (98). TGF-β1 binds to the C-terminal domain of TSP-5, and an additional binding site is the 3-repeat TSP in the presence of manganese. This combination increases TGF-β1-dependent transcription and enhances its bioactivity (98). Along with the activation of the TGF-β signal, the levels of TSP-5 are also elevated, which plays a critical role in skin fibrosis by promoting collagen deposition and modifying fibroblast functions (99, 100). Bone morphogenetic protein (BMP)-2 is a member of the TGF-β superfamily. *Yaoyao Du* et al. suggested that TSP-5 binds to BMP-2 via the C-terminus to prevent its interaction with the BMP-2 receptor, inhibit the osteochondrogenic transition of VSMCs, and improve vascular calcification (101).

### 4.3 ER stress

All three members of group B can active ER stress to regulate tissue development. TSP-4 is expressed in the heart and skeletal muscle during injury and damage, and it contributes to sarcolemmal stability and cardioprotection through binding to ATF6α-activated ER stress (102–104). TSP-4 is prominently localized within intracellular vesicles and the ER or its compartments in myocardial cells, with minor accumulation in the extracellular space (102). As a result of ER stress, TSP-4 KO mice infused with Ang II have exaggerated hypertrophic hearts and a high incidence of aneurysm but a protective role in endothelium-dependent relaxation in resistant arteries (105). TSP-3 is highly similar in structure to TSP-4 and is upregulated during cardiac disease. Although TSP-3 also activates ER stress by binding to ATF6α, TSP-3 oppositely enhances cardiac pathological conditions by inhibiting intracellular integrin and destroying myocardial membrane stability (85). The familial mutation in type 3 repeats of TSP-5 induces pseudoachondroplasia and multiple epiphyseal dysplasia resulting from pathological accumulation of mutated TSP-5 in the rough ER and apoptosis of the cells (106). Thus, mutated TSP-5 induces ER stress to regulate cartilage development.

### 4.4 NO signaling

TSP-4 promotes endothelial dysfunction and contributes to the process of hypertension by impairing NO bioavailability and blocking vascular vasodilation in Ang II-infused mice (105). TSP-5 plays a protective role in BP control by improving endothelium-dependent relaxation through CaKMII/eNO signaling. It binds to the C-terminus of Piezo1, which promotes intracellular Ca^2+^ influx, eNOS activation, and NO generation resulting from activation of endogenous Piezo1 currents (107). TSP-3 has not been found to regulate CVDs through NO signaling.

### 4.5 Immune cells

It is unknown whether TSP-3 acts on immune cells to affect the cardiovascular system. TSP-4 expression directly supports macrophage functions and switching of the proinflammatory phenotype during atherogenesis (108). Inflammation stimulates the expression of TSP-4 in macrophages, while increased TSP-4 promotes the accumulation and proinflammatory phenotypic differentiation of macrophages in LPS-induced peritonitis (108). In vascular inflammation, recombinant TSP-4 contributes to injury-induced restenosis by accelerating macrophage adhesion to VSMCs and increasing VSMC proliferation and migration (109). TSP-5 is also critical for inducing the beneficial phenotype of VSMCs and macrophages to help maintain vascular integrity and function (110, 111). The deletion of TSP-5 promotes VSMC migration and exacerbates VSMC calcification and atherosclerosis (111); moreover, this deletion results in a phenotypic shift of macrophages to atherogenic and osteogenic characteristics (112).

## 5 Conclusions and perspective

**Figure 2:**
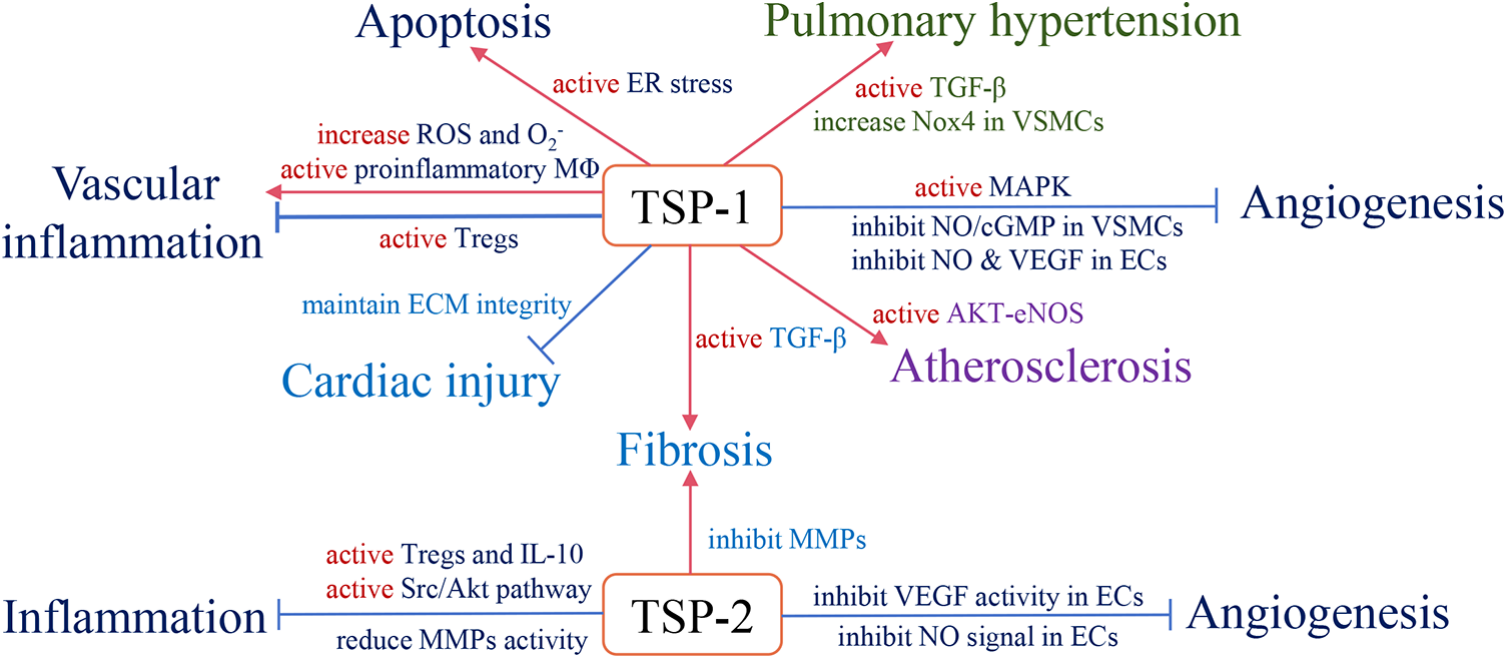
The role of subgroup A of the TSP family and relevant pathological mechanisms in the cardiovascular system. Red arrows define stimulatory effects, and blue arrows define inhibitory effects.

**Figure 3:**
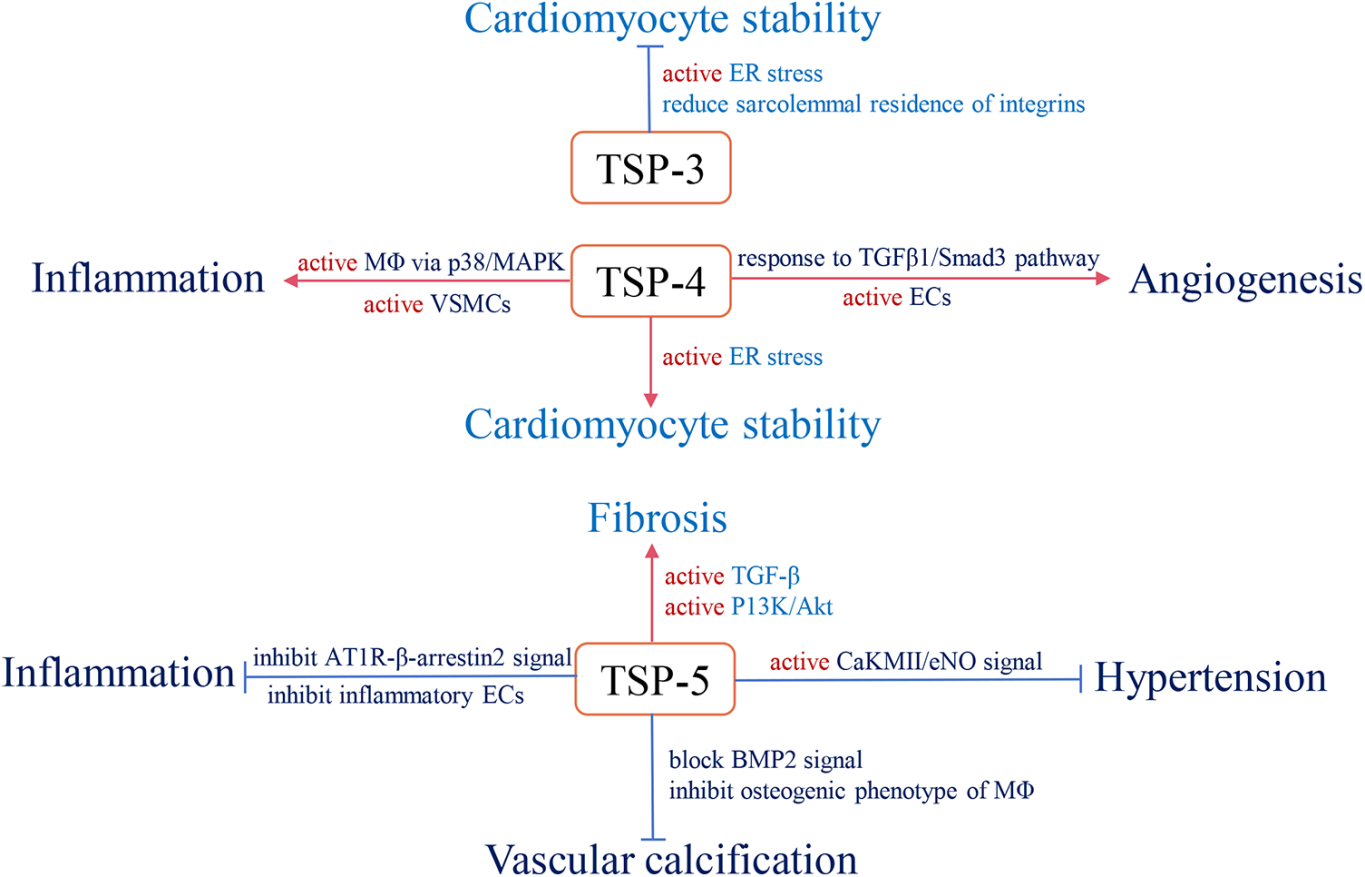
The role and relevant pathological mechanism of subgroup B of the TSP family in cardiovascular pathology. Red arrows define stimulatory effects, and blue arrows define inhibitory effects.

With long-term investigation, we gain a comprehensive understanding of TSP and its effective role in the cardiovascular system. Changes in circulating TSP production also affect the development and prognosis of clinical diseases. For instance, in group A, studies indicate that high circulating TSP-1 is associated with diabetic nephropathy, diabetic cardiovascular disease (113), and pulmonary hypertension (114). Elevated circulating TSP-2 is involved in coronary artery disease (CAD)-induced chronic heart failure (CHF) and increases patient mortality and risk of recurrent hospitalization (115). The group B members also participate in clinical diseases, but the related research is less than group A. Above all, we summarize the role of circulating TSP in clinical cardiovascular diseases (Table 1) and map the relative mechanisms of two TSP subgroups in the physiological and pathological processes of CVD (Figures 2, 3).

**Table 1:** Role of the TSP family members in clinical CVDs.

Diseases	TSP-1	TSP-2	TSP-3	TSP-4	TSP-5
CAV	Increase (35)	-	-	-	-
HF	Decrease	Increase (116)	-	-	-
		Increase in obese HfpEF (117)			
		High risk of HF in type 2 diabetes (118)			
		Increase in CAD induced CHF (115)			
Virus myocarditis	-	Increase (74)	-	-	-
PH	Increase (114)	-	-	-	-
DCM	-	-	-	-	Decrease (86)
MI	Increase	-	Decrease (119)	-	-
	High risk of AA in AMI (120)				
	Biomarker of thrombosis in STEMI (121)				
	Protective role of MACE risk in post-STEMI (67)				
AD	Increase (80)	Increase (122)	-	-	-
PAD	Increase (123)	-	-	Increase (124)	-
CHD	Increase (125)	-	-	-	Increase (126)
AS	Not change	Increase (127)	Not change	Not change	-
Atherosclerosis	-	Decrease (128)	-	-	-
AAA	Decrease (129)	Increase (130)	-	-	-
Diabetic complications	Increase in DM (131)	Increase in DKD (132)	-	-	-
	Independent risk factor of CAD in diabetes (133)				
Anemia	-	Increase (134)	-	-	-

CAV, cardiac allograft vasculopathy; HF, heart failure; HfpEF, heart failure with preserved ejection fraction; CAD, coronary artery disease; CHF, chronic heart failure; PH, pulmonary hypertension; DCM, dilated cardiomyopathy; MI, myocardial infarction; AA, atrial arrhythmias; AMI, acute myocardial infarction; STEMI, ST-segment elevation myocardial infarction; MACE, major adverse cardiac events; AD, aortic dissection; PAD, peripheral arterial disease; CHD, coronary heart disease; AS, aortic valve stenosis; AAA, abdominal aortic aneurysm; DM, diabetic myocardium; DKD, diabetic kidney disease.

TSP-1 and TSP-2 have significant antiangiogenic effects because of type I repeats (135). ABT-510 is a nonpeptide analog of the type I repeat and is constructed with a single D-amino acid substitution that results in 1,000-fold antiangiogenic activity (136). ABT-510 inhibited VEGF-induced microvascular EC migration and exerted strong antiangiogenic effects to prevent graft arteriosclerosis in rats (137); moreover, ABT-510 induced inflammation in mice with inflammatory bowel disease (138). In humans, the use of ABT-510 alone or in combination with other therapies for the treatment of advanced parenchymal or epithelial carcinoma has been tested in phase I and II clinical trials (139–141). Several studies have shown that ABT-510 is safe and efficient at restraining tumor growth and inhibiting tumor neovascularization, and combination therapy increases the efficiency of anticancer therapy (142–144).

The TSP/CD47 axis plays a vital role in many pathological processes in the cardiovascular system. Preclinical trials of blocking CD47 with monoclonal antibodies in animal ischemia models have shown improved angiogenesis and a significant increase in tissue survival (39, 145). The CD47 antibody (CC-90002) has been tested for biosafety/tolerability in phase I clinical trials and beneficial effects for neoplastic hematologic disorders, while another humanized anti-CD47 monoclonal antibody (Hu5F9-G4) may be beneficial for acute myeloid leukemia and advanced solid malignancy (146). The activated TGF-β pathway has strong profibrotic activity, the activation sequence (LSKL) in TSP-1 was mapped, and an LSKL peptide was developed for competitive binding (22). The LSKL peptide blocks TGF-β release and inhibits fibrosis in various animal models, such as diabetic nephropathy (147), liver fibrosis (148), and skin scarring (149). However, in Ang II-infused apolipoprotein E-deficient mice, decreased activity of the TGF-β pathway promotes AAA and atherosclerosis (150). Thus, antifibrotic therapy has dual effects on different pathological conditions, concentrating on preventing advanced parenchymal organ fibrosis or local treatment, which may be potential therapeutic directions.

Numerous studies have shown that patients with CVD have a greater cancer risk than do healthy individuals. There are connections between cancer and atherosclerotic cardiovascular disease (ASCVD) (151), myocardial infarction (152) and heart failure (153). Thus, a new discipline called “reverse cardio-oncology” was established, and studies need to identify the shared mechanisms and pathways between these 2 diseases (9). We summarize the shared mechanisms and pathways of the TSP proteins in cardiovascular disease and cancer in Table 2. Thrombospondins strongly regulate angiogenesis, tissue fibrosis and inflammation via effects on the cardiovascular system and tumor microenvironment. TSP-1 is involved in myocardial fibrosis and tumor growth by activating the TGF-β pathway (22, 160). TSP-4 mediates inflammatory macrophage infiltration, which not only exacerbates atherogenesis in the vasculature but also induces breast cancer cell growth in mice (89, 161). ABT-510, a type I repeat of TSP-1/TSP-2, can prevent graft arteriosclerosis in animal models (137) while inhibiting tumor neovascularization as an antineoplastic drug (139). Although there is no direct evidence that TSPs participate in the crosstalk between CVD and cancer, we reasonably hypothesize that this occurs.

**Table 2:** Common mechanisms of TSP in CVD and cancer.

TSPs	CVD	Cancer	Common mechanism
Group A	Promotes myocardial fibrosis	Promotes tumor growth	TSP1-TGF-β
	Promotes endothelial dysfunction	Promotes tumor-initiating stem cells in hepatocellular carcinoma (154)	TSP1-CD47-(SIRPα)
	Anti-angiogenesis	Inhibit tumor-associated angiogenesis (155, 156)	TSP1/2-CD47/CD36/integrin
			TSP1-NO signal (157)
Group B	Aggravates atherogenesis	Promotes breast cancer cell growth	TSP4-Macrophage
	Pro-angiogenesis	Promotes gallbladder cancer growth (158)	TSP4-integrin α2
	Pro-angiogenesis	Promotes breast cancer growth	TSP4-TGF-β1
	Reduce blood pressure	Anti-apoptosis of prostate cancer (159)	TSP5-Ca^2+^
	Anti-atherogenic effect	Promotes prostate cancer progression	TSP5-integrin

TSP-5 has been shown to play a protective role in the cardiovascular system and affects conditions, including hypertension, atherosclerosis, and AAA. TSP-5 helps sustain the contractile phenotypes of VSMCs. It has been demonstrated that TSP-5 deficiency induces VSMC migration while aggravating VSMC calcification and atherosclerosis (111); moreover, the angiotensin II type 1 receptor/β-arrestin-2 signaling pathway is also activated by the absence of TSP-5, resulting in a high risk of AAA (162). TSP-5 supplementation is a potentially effective therapy for CVD treatment. However, TSP-5 also plays a critical role in the migration and invasion of various cancer cells and is a potential target for cancer treatment (163). Cancer patients may benefit from systematic administration of anti-TSP-5 therapy while under the threat of CVD. In conclusion, local targeted therapy or treatment that focuses on interactions with TSPs and signaling pathways may be meaningful (112).
